# Methadone associated long term hearing loss and nephrotoxicity; a case report and literature review

**DOI:** 10.1186/s13011-019-0236-z

**Published:** 2019-11-06

**Authors:** Saeedeh Ghasemi, Shadi Izadpanahi, Mohammad Ali Yaghoubi, Jeffrey Brent, Omid Mehrpour

**Affiliations:** 1 Medical Toxicology and Drug Abuse Research Center (MTDRC), Birjand University of Medical Sciences, Ghaffari Avenue, Vali-Asr hospital, Birjand, Iran; 2 Student Research Committee, Birjand University of Medical Sciences, Birjand, Iran; 3 Metabolic Syndrome Research Center, Mashhad University of Medical Sciences, Mashhad, Iran; 4 Department of Medicine, University of Colorado School of Medicine, Aurora, CO USA; 5 Rocky Mountain Poison and Drug Safety, Denver Health and Hospital Authority, 1391 Speer Blvd, Denver, CO 80204 USA

**Keywords:** Rhabdomyolysis, Hearing loss, Methadone, poisoning

## Abstract

**Background:**

Methadone is a long-acting opioid receptor agonist. Reported adverse effects of methadone include constipation, respiratory depression, dizziness, nausea, vomiting, itching, sweating, rhabdomyolysis, QT prolongation, and orthostatic hypotension. Hearing loss has been rarely reported following methadone use, and when reported, long term follow-up is rare.

Herein we report a case of methadone poisoning with rhabdomyolysis, acute kidney injury, and persistent hearing loss documented by a 2 year follow up.

**Case presentation:**

The patient was a 34 years old male who presented with a reduced level of consciousness and acute hearing loss after suicidal ingestion of 40 mg of methadone while experiencing family-related stresses. He had no prior history of methadone use, abuse, or addiction.

Initial laboratory testing was significant for a serum creatinine concentration of 4.1 mg/dl, a mixed metabolic and respiratory acidosis, thrombocytopenia, abnormal hepatic transaminases, and coagulation tests. The patient then developed severe rhabdomyolysis. Also, audiometry showed a bilateral sensorineural hearing loss. The patient required hemodialysis for 11 days while his metabolic abnormalities gradually resolved. However, his hearing loss was persistent, as demonstrated by 2 years of follow up.

**Conclusion:**

Our patient simultaneously had kidney failure, rhabdomyolysis, and permanent hearing loss following methadone poisoning. Although rare, ototoxicity and permanent hearing loss may happen in cases of methadone poisoning. While opioid-induced hearing loss is uncommon, methadone toxicity should be taken into account for any previously healthy patient presenting with acute hearing loss with or without rhabdomyolysis.

## Background

Methadone is a synthetic μ-opioid receptor agonist used in the treatment both of pain and opioid dependence [1–4]. In the last decade, the amounts of opioids prescribed, and the consequent death rate and abuse of these drugs, has dramatically increased [5].

Rapidly progressive sensorineural hearing loss has been rarely reported in opioid analgesic users, including those using methadone [6–8]. The mechanism of this hearing loss is not well understood. Suggested causes include ischemia [9], genetic predisposition [10], direct cochlear toxicity [11], and hypersensitization that manifests upon re-exposure after a period of opioid withdrawal [12, 13]. Most of the reported cases recover after a period of opioid abstinence [11]. Although hearing loss due to methadone poisoning is rare, it is generally transient [14]. Apparent permanent hearing loss due to methadone poisoning is infrequent [14]. However, for the reported cases there is often a lack of long-term follow-up. Here, we report a case of long-term follow-up of a patient with permanent bilateral sensorineural hearing loss after a methadone overdose complicated by rhabdomyolysis and severe acute kidney injury.

## Case presentation

A 34-year-old male presented to the Emergency Department with a reduced level of consciousness and hearing loss 21 h after ingesting a 40 mg methadone tablet in a suicide attempt in front of his family after a serious conflict with his father. The family denied any history of a psychiatric disorder or prior methadone use. Prehospital emergency service administered 0.4 mg of naloxone due to apneustic breathing and miotic pupils, which resulted in a marked improvement in the patient’s breathing. He and his family denied any specific drug abuse history. He smoked cigarettes but did not use alcohol. Before this suicide attempt, he was healthy without any significant past medical history and did not have any known auditory deficits.

On admission to the Emergency Department (ED) the patient was lethargic, had miotic pupils and the following vital signs: blood pressure 110/70 mmHg, heart rate 110 beats per minute, respiratory rate 16 breaths per minute, temperature **37**^**0**^ C, oxygen saturation while receiving 8 l oxygen per minute by nasal cannula 89%. At the time of admission to the ED and he complained of bilateral hearing loss. His urine was noted to be dark, and a rapid urine toxicology screen test was positive for methadone but was negative for amphetamines, barbiturates, benzodiazepines, cocaine, morphine, phencyclidine, and tetrahydrocannabinol. Blood acetaminophen, salicylate, and ethanol assays were negative.

Initial laboratory studies showed a mixed metabolic and respiratory acidosis (pH 7.11, PCO2 41.8 mmHg, HCO3 13.5 mmol/l), aspartate aminotransferase (AST) 1417 U/L, alanine aminotransferase (ALT) 1125 U/L, BUN 85 mg/dL, Cr 4.1 mg/dL, international normalized ratio (INR) 2.24, and Platelet count of 66,115 /mm^3^ (Table 1). The microscopic examination of his peripheral blood smear was normal.

**Table 1:** Laboratory finding during admission of case

Day of admission	1	2	3	4	5	7	8	9	10	13	15
WBC	10,150	14,116		10,400	7300	12,700	13,910		14,150	10,300	6210
PLATELET	66,115			110,000					150,405	192,000	180,300
CPK (U/L)		97,231		77,150	20,400	6087	1456	1389	803	556	120
Alkaline Phosphatase (U/L)	330			234		361	427	303	265	300	180
PT (Second)	22	20.1	16.8	13	14	13.8	15.5	18	15.6	17	14
PTT (Second)	37	25	31.7	26	24	27.5	25	32	32	29	25
INR	2.24	1.91	1.42	0.94	1.06	1.02	1.26	1.6	1.26	1.5	1.1
BUN (mg/dL)	85	155	170	245	276	206	151	130	170	90	24
Cr (mg/dL)	4.1	6.2	7.1	9.1	10.8	9.8	8	6.6	3.8	2	1.4
AST (U/L)	1417		1800	1415	670	220	180	110	80	90	43
Uric Acid (mg/dL)	11		11	8			7		6	6	3
ALT (U/L)	1125		1203	1610	500	480	310	179	115	45	30

*Abbreviations*: *WBC* White blood cell, *CPK* Creatine phosphokinase, *PT* Prothrombin time, *PTT* Partial thromboplastin time, *INR* International normalized ratio, *BUN* Blood urea nitrogen, *Cr* Creatinine, *AST* Aspartate aminotransferase, *ALT* Alanine aminotransferase

Initial treatment consisted of the administration of intravenous fluids and sodium bicarbonate. Twelve hours post-admission, the patient was transferred to the Intensive Care Unit (ICU), where he received a naloxone infusion for 3 days.

On admission to the ICU, SPO2, while using an oxygen mask at 8 l/minute was 97%, and his arterial blood gas assay showed: pH 7.33, PCO2 43.1 mmHg, and serum HCO3 23 mmol/l.

**Fig. 1:**
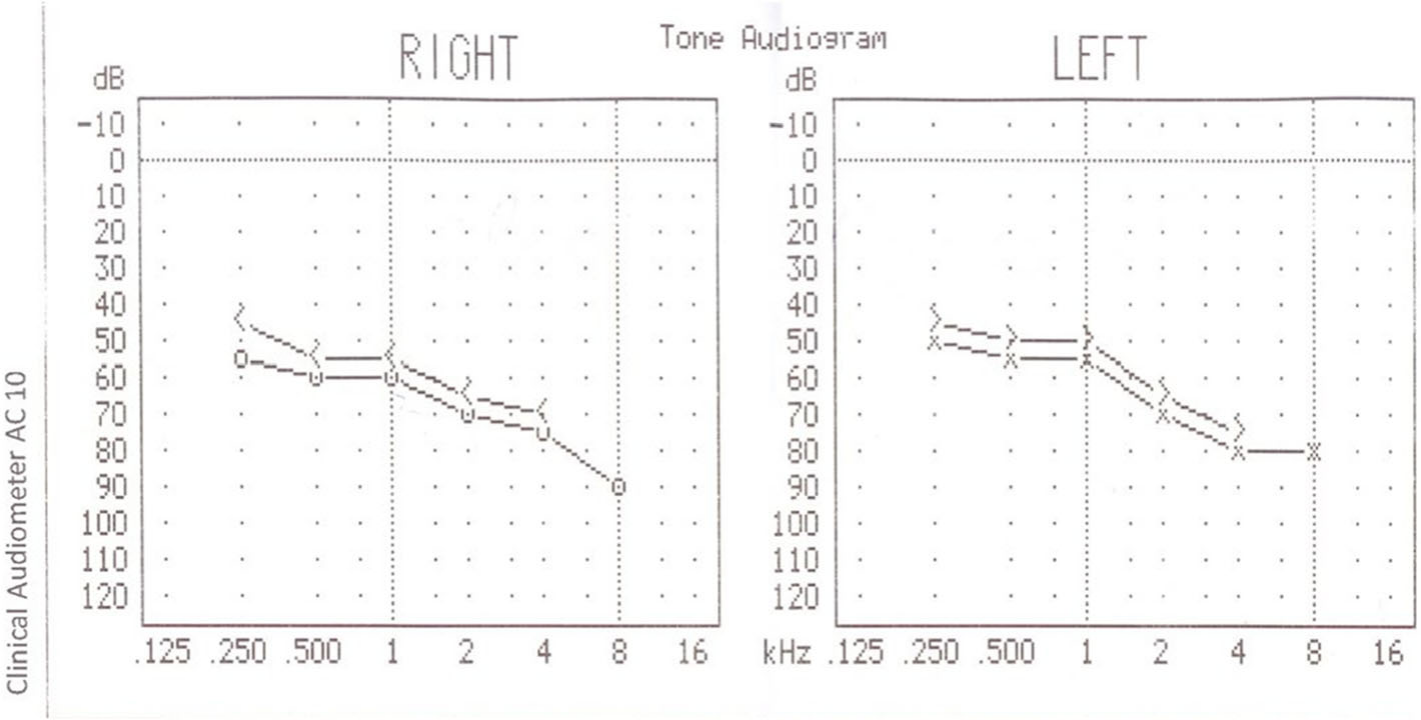
Audiometry performed on the patient on the second day of admission

On the following day, he was alert and breathing normally with a leukocytosis of 14,116 cells/microliter (mcl) (Table 1), but no evidence of infection or fever. No antibiotics were administered. His creatine phosphokinase (CPK) was found to be 97,231 U/L. He continued to demonstrate hearing impairment and audiometry showed a bilateral sensorineural hearing loss (Fig. 1).

By the third day of admission, the patient complained of myalgia and had a urine output of approximately 100 ml/24 h, a BUN of170 mg/dL, serum creatinine of 7.1 mg/dL, and a serum uric acid 11 mg/dL. Non-heparinized hemodialysis was initiated. Kidney sonography was suggestive of parenchymal kidney disease and acute tubular necrosis. He required hemodialysis for 11 days.

During 10 days of admission to the ICU, his hepatic transaminases and CPK gradually normalized (Table 1).

The patient was transferred to the internal medicine ward on day 11. During the 5 days of hospital stay in the internal ward, the patient’s hearing loss continued despite the normalization of his laboratory studies. He was discharged after 15 days of hospitalization. After 2 years of follow-up, his hearing loss persisted.

## Discussion

Here we report a case of methadone poisoning following a suicide attempt with a 40-mg tablet of methadone who exhibited rhabdomyolysis, acute renal failure, and chronic bilateral sensorineural hearing loss. Unlike most prior cases, our patient’s hearing loss persisted despite the normalization of his renal function and resolution of his metabolic abnormalities.

Sudden sensorineural hearing loss has several potential causes, including infections, autoimmune diseases, trauma, vascular disorders, and neoplastic diseases, but most cases are idiopathic [15]. Many of these cases are caused by autoimmune processes or, in rare cases, medications, including some antibiotics (such as aminoglycosides), diuretics, chemotherapeutic agents, and anti-inflammatory drugs [16, 17].

Ototoxicity is a known rare complication of opioid use. In 1979, Mulch et al. reported the first case of opioid-induced hearing loss, which was associated with hydrocodone abuse. Since then, several case reports have described both reversible and irreversible bilateral hearing loss following hydrocodone/acetaminophen [6], oxymorphone [11], propoxyphene [18, 19] and heroin abuse [12, 20–23]. Hearing loss following methadone use has been occasionally reported [8]..

In 2009, the first case of bilateral hearing loss due to methadone was reported in a 37-year-old man who overdosed on 75 mg methadone tablets [24]. Thier patient, with no previous history of an auditory disease, had hearing loss and tinnitus, which resolved in 10 days.

Subsequently, a number of patients have been reported to develop hearing loss after methadone use [8, 13, 16, 24, 25]. Most of the reported cases of methadone-induced hearing loss were transient and recovered within t 10 days of ingestion [13, 24, 25]. Very few cases reported long term hearing loss after methadone ingestion [8, 16]. Vorasubin et al. [8] first reported persistent bilateral sensorineural hearing loss following methadone ingestion in a 23 years old man, which after 9 months of follow up was deemed permanent. Saifan et al. [16] reported severe sensorineural hearing loss in a 31-year-old male who showed a persistent hearing loss after 2 months of follow up. He was prescribed binaural hearing aids. Our case had 2 years of follow up. Given the lack of resolution during this period, it is likely that his hearing loss is permanent.

The mechanism through which opioids cause hearing loss has not been adequately explained. Among the theories that have been offered for this complication is the contamination of the drug with unknown materials. However, this theory is more likely for heroin-induced sensorineural hearing loss, since methadone is usually produced in pharmaceutical laboratories [16].

Another theorized mechanism for opioid-associated hearing loss is hypoperfusion of the vestibulocochlear system secondary to opioid-induced vasospasm or vasculitis. The origin of this theory is the observation of cerebral infarcts following heroin used due to the resulting vasculitis [8, 22]. However, here too, vasculitis from a pharmaceutical preparation is less likely. Alternatively, genetic polymorphism in drug metabolism has been postulated to cause opioid-induced hearing loss. This theory posits that some people are prone to hearing loss following opioid use and may even experience it after the usual therapeutic doses of opioids, while others develop hearing loss only after taking higher doses of opioids [8]. Another theory of this disorder is the direct cochlear damage by agonism at opioid receptors. Animal studies have indicated that opioid μ, δ, and κ receptors have been found on the cochlea [26, 27]. Poisoning with opioids leads to over-stimulation of the kappa receptor and decreases the activity of the cochlear hair cells [28]. Also, when the μ receptors in the cochlea bind to their agonists, they decrease the activity of adenylate cyclase and alter signal transduction [8].

Our patient had metabolic acidosis, rhabdomyolysis, and acute renal failure requiring hemodialysis. Rhabdomyolysis is a serious syndrome caused by the breakdown of skeletal muscle fibers followed by leakage of muscle contents into the patient’s circulation [29]. The causes of rhabdomyolysis include the traumatic and non-traumatic injury of muscles, drugs, toxins, infections, and electrolyte disturbances [30], but the development of rhabdomyolysis following opioid poisoning has only been reported in a few cases [28–31]. The hypotheses that have been raised for the cause of rhabdomyolysis in opioid users include dehydration, vascular insufficiency, muscle contraction, vasospasm, shock, trauma, seizure, acidosis, respiratory failure, and direct effects of the opioid [3, 25]. Concomitant of rhabdomyolysis and hearing loss following opioid poisoning as in our patient was rarely reported [28].

Surprisingly, rhabdomyolysis and acute kidney injury were in the first day after ingestion in our case. Rapid onset of rhabdomyolysis and acute kidney injury following methadone poisoning has rarely reported [31, 32]. Likely, coma, immobilization, volume depletion and direct effect of methadone on the kidney were responsible. Similar to our case, Saifan et al. [16] reported hearing loss still present 2 months after concomitant with transient kidney failure after methadone ingestion. The relationship between these complications is unclear, but the similarity between the channels and the junctions between stria vascularis and kidney tubules is a possible explanation [8]. Further studies may more accurately identify the mechanisms that lead to these complications and their predisposing causes.

## Conclusion

Methadone poisoning may induce rhabdomyolysis, acute kidney injury, and permanent hearing loss. Although most ototoxicity due to methadone poisoning is manifested as transient hearing loss, permanent hearing loss may rarely occur. While opioid-induced hearing loss is uncommon, methadone toxicity should be taken into account for any previously healthy patient presenting with acute hearing loss, with or without rhabdomyolysis. The relationship, if any, between rhabdomyolysis and hearing loss, warrants further study.

## Data Availability

The dataset used and analyzed during the current study is available from the corresponding author on reasonable request.

## Abbreviations

ALT: Alanine aminotransferase
AST: Aspartate aminotransferase
BUN: Blood urea nitrogen
CPK: Creatine phosphokinase
Cr: Creatinine
INR: International normalized ratio
PT: Prothrombin time
PTT: Partial thromboplastin time
WBC: White blood cell
